# Computational approaches to selecting and optimising targets for structural biology

**DOI:** 10.1016/j.ymeth.2011.08.014

**Published:** 2011-09

**Authors:** Ian M. Overton, Geoffrey J. Barton

**Affiliations:** a MRC Human Genetics Unit, Institute of Genetics and Molecular Medicine, Western General Hospital, Crewe Road, Edinburgh EH4 2XU, United Kingdom; b College of Life Sciences, University of Dundee, Dundee DD1 5EH, United Kingdom

**Keywords:** MSA, Multiple Sequence Alignment, PTM, Post Translational Modification, SSPF, Scottish Structural Proteomics Facility, MCC, Matthew’s correlation coefficient, AROC, Area Under the Receiver Operator Characteristic curve, Target selection, Crystallisation, Structural genomics, Structural biology, Bioinformatics, Construct design

## Abstract

Selection of protein targets for study is central to structural biology and may be influenced by numerous factors. A key aim is to maximise returns for effort invested by identifying proteins with the balance of biophysical properties that are conducive to success at all stages (e.g. solubility, crystallisation) in the route towards a high resolution structural model. Selected targets can be optimised through construct design (e.g. to minimise protein disorder), switching to a homologous protein, and selection of experimental methodology (e.g. choice of expression system) to prime for efficient progress through the structural proteomics pipeline.

Here we discuss computational techniques in target selection and optimisation, with more detailed focus on tools developed within the Scottish Structural Proteomics Facility (SSPF); namely XANNpred, ParCrys, OB-Score (target selection) and TarO (target optimisation). TarO runs a large number of algorithms, searching for homologues and annotating the pool of possible alternative targets. This pool of putative homologues is presented in a ranked, tabulated format and results are also visualised as an automatically generated and annotated multiple sequence alignment. The target selection algorithms each predict the propensity of a selected protein target to progress through the experimental stages leading to diffracting crystals. This single predictor approach has advantages for target selection, when compared with an approach using two or more predictors that each predict for success at a single experimental stage. The tools described here helped SSPF achieve a high (21%) success rate in progressing cloned targets to diffraction-quality crystals.

## Highlights

► Identifies key considerations in target selection and optimisation. ► Approaches to assign useful protein features and structure/function relationships. ► Comparison of latest crystallisation propensity predictors on nonredundant data. ► Discusses single point of reference target selection/optimisation resources. ► Guidance on using the SSPF Target Optimisation Utility (TarO).

## 1 Introduction

Of all techniques applied in molecular biology, macromolecular crystallography reveals the most exquisite details about the machines of life. Advances in X-ray sources, computational methods and cryo-techniques over the last 20 years have led to a dramatic increase in the rate at which a protein structure may be determined once diffracting crystals have been obtained. Unfortunately, expressing proteins at levels suitable for structural studies and obtaining crystals that diffract remain the major bottlenecks in most structural biology laboratories [1–3]. Accordingly, computational sequence analysis and similar methods are often applied to increase the chances of success. Common strategies are to seek out related proteins that might fare better than the preferred target (e.g. orthologues, pathway members), to “optimise” the target protein in some way, or to adjust the laboratory approach (e.g. choice of expression system) [3–13]. If the native protein fails to crystallise, optimisation typically starts with truncation of the protein into likely domains, or the removal of disordered regions, but may include more sophisticated engineering. These strategies rely on the application of computational tools for sequence analysis and alignment in conjunction with the structural biologist’s experience. Although a single investigator might spend days studying options to try on their protein, in a high-throughput structural proteomics environment it is necessary to streamline this process by introducing a higher degree of automation. In this article, we examine computational approaches for selecting and optimising proteins for crystallography with emphasis on those developed at the University of Dundee [12,14–16] as part of the Scottish Structural Proteomics Facility (SSPF) [3]. Although developed with high-throughput crystallography in mind, most of the tools described here are equally applicable to smaller-scale structural studies.

## 2 Influence of project scope on structural proteomics target selection

The overall approach to selecting targets is dictated by the scope of the project. In the subsections below we outline a few examples of how the research aims may impact on target selection and optimisation. A common principle in target selection is to identify proteins (e.g. orthologues) that both satisfy the project aims and are relatively amenable to structural characterisation. Target optimisation is applicable to almost every project and is discussed in greater detail in Section 6 of this article. Indeed analyses enabled by the Target Optimisation Utility (TarO) [12], such as prediction of domain boundaries, are useful in any structural biology laboratory – even for work that focuses specifically on a single target. Further information on current structural proteomics projects can be obtained by exploring links from the International Structural Genomics Organisation (ISGO) list of active initiatives [17].

### 2.1 Structural proteomics on a specific organism

Some projects seek to provide structural coverage across the whole proteome of a particular organism, such as *Thermotoga maritima* [18] or *Saccharomyces cerevisiae* [10,19]. This kind of genome-wide approach rules out searching for more tractable orthologues; however, ranking targets according to their predicted success may inform experimental strategy. Optimisation of the construct sequence may also be productive, for example to minimise protein regions predicted to be disordered or to adjust codon usage [11,12,20]. Such optimisation can be useful for all targets, but is more often adopted as a salvage strategy for targets that flounder with a standard approach.

### 2.2 Structural biology projects for drug discovery and biological chemistry

Some structural proteomics projects focus on targets that might be suitable in drug discovery against a specific pathogen, for example, the *Mycobacterium tuberculosis* structural genomics consortium [21]; even these consortia may have scope for flexibility across different targets amongst pathways and sub-networks. However, prioritisation of druggable targets with favourable properties, such as control of metabolic flux and therapeutic selectivity, limits the choice of alternative structural targets and constructs [22]. Structural characterisation of a biological pathway or a particular enzyme function enjoys greater flexibility, where exploration of different orthologues and constructs (e.g. the catalytic domain) may be helpful.

### 2.3 Mapping protein structure space

Efforts to extend protein structure space coverage (e.g. [7,23]) have good scope for selecting the most favourable candidates from groups of structurally similar proteins, at least where structural relationships can be reliably inferred. Similar flexibility is available to efforts that focus on particular classes of proteins (e.g. [24,25]). As noted above, target optimisation is also useful in these contexts.

## 3 Useful protein features in target selection and optimisation

In order to identify favourable targets and constructs, significant attention has been given to exploring biophysical properties and investigating protein selection strategies that correlate with success in obtaining a structure (e.g. [9,14–16,26–30]). To give a few examples, properties influencing soluble expression include isoelectric point (pI), hydrophobicity, and sequence length; properties influencing production of diffraction-quality crystals from purified protein include surface entropy, disordered sequence, and protein post-translational modifications [11,26–32]. Many of these features, as well as relevant algorithms and databases are summarised in Table 1. Properties that impact on success are often correlated. For example, regions that participate in protein–protein interactions have greater hydrophobicity [33,34], and sites of post-translational modification are enriched for disordered regions [31]. In addition, individual biophysical properties have been shown to significantly influence multiple pipeline stages. For example, hydrophobicity affects soluble expression, purification and crystallisation; glycosylation affects soluble expression and crystallisation; while the sequence length has an impact on cloning, soluble expression and crystallisation [11,27]. Moreover, selection or engineering for success at a given experimental stage can hinder progress at other parts of the structure determination pipeline. For example, surface entropy and charge are related because several high entropy residues have charge (e.g. Lys, Glu, Arg). In general, more surface charge, and consequently higher entropy, favours solubility; on the other hand lower surface entropy, and consequently charge, favours crystallisation [28,35]. Therefore, target selection and optimisation would ideally find protein chains that possess the correct balance of properties required for successful progression through all experimental stages leading to a high-resolution structural model. Indeed, algorithms have been developed with this goal in mind [14–16,36]. Algorithms are also available to predict progression at a particular pipeline stage [28–30,37]; for example PXS aims to predict the crystallisation of ‘well-behaved’ soluble proteins [28]. Section 5, below, gives further discussion of these and other tools.

An assessment of the existing functional annotation available to inform structure interpretation is also useful for target selection. Indeed, new structures are difficult to interpret without some functional knowledge, and so make a less immediate contribution to biological understanding in the scientific community. For example, target selection in the SSPF included a score to estimate functional annotation based on the Gene Ontology [3,38].

Correlates of success are less understood for integral membrane proteins, which represent around 25% of protein-coding genes [39] but only 1% of proteins with high resolution structures [40,41]. Indeed, membrane protein expression, purification and crystallisation are individually very challenging [42,43]. However, membrane proteins are relatively amenable to computational characterisation, partly due to physical constraints imposed by the phospholipid bilayer. Indeed, existing approaches (e.g. TMHMM2 [44], Phobius [45]) perform well in predicting membrane protein topology, including identification of cytoplasmic and extracellular regions. Current selection strategies seek to enhance membrane protein tractability by avoiding protein disorder and hetero-oligomeric complexes [25]. Intrinsically disordered proteins are also thought to represent a significant proportion of protein-coding genes and are resistant to structural studies; these multifunctional proteins adopt different conformations according to protein interactions, environmental conditions (e.g. pH, temperature) and small changes in amino acid sequence [46–48]. Protein complexes are also typically difficult to work with, and have been a specific focus of the European ‘SPINE2 complexes’ initiative [49]. Target selection methods have been developed to identify “low-hanging fruit” for crystallography of protein complexes, however this area remains challenging [5,13]. Protein interaction databases, such as MINT [50] and PIPS [51] enable inference of complexes, for inclusion or exclusion of candidate targets. This article does not detail approaches for these especially difficult classes of targets.

## 4 Assignment of protein structure and function relationships

A fundamental technique in target selection is to estimate the relationships in structure and function between the target and other proteins. This approach allows alternative structural candidates to be identified (e.g. orthologues), as well as supporting efficient coverage of protein structure space [7,23]. In practice, automated sequence searching is a crucial component for inferring similarity in structure and/or function across genomes [52,53]. For example, sequence similarity to human proteins provides a coarse filter against unsuitable drug targets, or alternatively an inclusion criterion for targets relevant to human biology. The ‘Rost curve’ [54] is a heuristic for the estimation of protein structural relationships from sequence alignments and provides a formula that combines alignment length and percentage identity/similarity. Target selection pipelines have employed the ‘Rost curve’ as a means to obtain proteins that are expected to be structurally similar to a candidate target in order to: (A) add expected structurally similar proteins to a pool of candidate targets and (B) determine if structural information already exists, leading to target deprioritisation or alternatively to suggest models for phasing by molecular replacement [3,6,12]. Importantly, the ‘Rost curve’ is algorithm-dependent and therefore requires calibration, as has been done for SSEARCH [6].

Searching orthologous sequence databases (e.g. eggNOG [55], InParanoid [56]) can be a productive strategy to expand the possible pool of targets available for consideration. One approach examined structural similarity to orthologous groups using the Rost thresholds in order to include the group of putative orthologues into the pool for further study [12]. However, sequence homology for non-globular protein segments, such as transmembrane regions and signal peptides, requires careful consideration because sequence relationships in these regions frequently reflect convergent evolution due to physical constraints [57]. Sequence-based approaches to infer relationships in protein structure and function were largely developed from studies of globular proteins and therefore may not translate appropriately to other protein classes, even when low-complexity filtering is applied [57]. Visual inspection of an annotated multiple sequence alignment (MSA), including examination of sequence feature conservation, is invaluable for assessment of structural and functional similarity [12]. However, construction of a MSA is not necessarily straightforward (e.g. for multi-domain proteins), and so rounds of manual interpretation and realignment may be required. Informative features for this purpose include protein disorder [58–60] secondary structure [61], domains [62,63], motifs [64] and post-translational modifications (e.g. [65–67]).

Automatically identifying and deselecting a target when significant progress has been made by a different research group is an important aspect of structural proteomics work so that effort is not wasted [8]. Information sharing is crucial in target deselection, which is typically based on regular searches of TargetDB, PepcDB and PDB [68,69]. Tools with capabilities relevant to target deselection include PiMS [70] and SeqAlert [4]. In order to reduce duplication of effort, the USA Protein Structure Initiative production phase (PSI-2) has integrated target selection bioinformatics across its four large-scale centres [23].

## 5 Predicting success in the structural proteomics pipeline

Having identified a pool of sequences that possess appropriate structure and function relationships according to the project scope, the next logical step is to determine promising candidates for experimental work. As noted above, successful progression of a selected target through to the stage of diffraction-quality crystals is a critical consideration. Algorithms to estimate this include XANNpred, OB-Score, ParCrys, XtalPred, PPCpred and PDPredictor [14–16,36,71,72]. Approaches focused on key stages of the structural biology pipeline have also been developed, including predictors of soluble expression (e.g. PROSO [29], SOLpro [37]) and crystallisation (e.g. PXS [28], SECRET [30]). SECRET is limited to only accept sequences of length 46–200 residues [30]. Predictors that focus on a specific experimental stage are particularly useful when protein targets have already reached the given stage in the pipeline, especially in target optimisation to propose alternative constructs; the SERp surface entropy reduction server is an example [9]. Estimating overall success of selected targets with a single predictor is much more appealing than using multiple single-stage predictors. Indeed, a linear combination of multiple predictors suffers from error multiplication and makes candidate target ranking more cumbersome. Consider a strategy combining two predictors to separately estimate soluble expression and crystallisation propensity. If each predictor gave 75% accuracy individually, accuracy for progression through both stages would be only 56%. Moreover, biophysical properties that are advantageous at one stage (e.g. solubility) may conflict with properties required for success at another stage (e.g. crystallisation) [11,28,35]. Accordingly, an attractive approach applies a single algorithm to select targets with the right balance of biophysical properties to successfully navigate all stages of the structural proteomics pipeline. As noted above, this strategy is available *via* the algorithms XANNpred [16,73], PPCpred [72,74], PDPredictor, XtalPred [36,75,76] and ParCrys/OB-Score [14,15,77]. Interestingly, PPCpred provides a single prediction for overall success, as well as estimating success at three individual pipeline stages [72] and so informs on expected point(s) of failure. The subsections below give further discussion on the relative merits of these methods, with emphasis on those developed at the Scottish Structural Proteomics Facility (SSPF). Of the algorithms examined, XANNpred was found to be best-performing (Subsection 5.4 and [16]).

### 5.1 The OB-Score

**Fig. 1:**
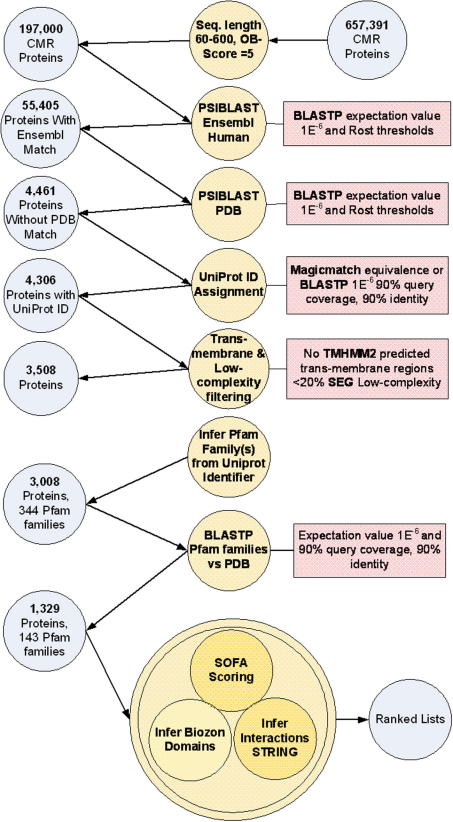
An example target selection pipeline. This figure summarises a target selection project conducted in the SSPF, starting with the Comprehensive Microbial Resource (CMR) database [94] in order to identify tractable targets that were in novel structure space and structurally similar to human proteins. Circles on the left-hand side represent proteins, circles in the middle electronic analysis and rectangles give selection thresholds. The SOFA (specificity of functional annotation) scoring [3] provided an estimate of available functional annotation on the candidate targets. The analyses in this pipeline were run within customised scripts developed at the SSPF. Following manual inspection, targets selected from the ranked lists were analysed using TarO.

The OB-Score built upon findings about the correlation between hydrophobicity, isoelectric point (pI) and crystallisation success in the *T. maritima* proteome [26]. Three clusters were identified; cluster A which contained 75% of crystallised proteins and 60% of the *T. maritima* proteome, cluster B (27% crystals, 20% of proteome), and cluster C (a single crystal, 10% of proteome) [26]. The OB-Score provides a measure of similarity for a protein’s pI and hydrophobicity to that of previously crystallised proteins in the PDB [15]. Briefly, a redundancy-filtered set of PDB structures and UniRef100 [78] provided the basis for developing a Z-score matrix, and validation was performed against available information from structural proteomics consortia [15]. Software to calculate the OB-Score is available for download from [79] and predictions are also available from a webserver [77]. Notably, the OB-Score is fast to calculate and therefore easily applied to large datasets, within a multi-criterion target selection pipeline as shown in Fig. 1.

### 5.2 ParCrys

ParCrys extends the number of features considered in the OB-Score and implements them within a non-parametric statistical framework to estimate a density function from PDB structures without requirement for negative examples [14]. Parameterising the density function only on a set of positive examples has the advantage of avoiding complications around defining ‘non-crystallisable’ targets. Feature selection was done with public data from structural proteomics consortia [68] to identify single amino acid frequencies (S, C, G, F, Y, M) as predictive features additional to hydrophobicity and isoelectric point. Therefore, ParCrys represents a more sophisticated algorithm than the OB-Score and was found to perform well on several non-redundant blind test datasets, including specific construct sequences taken from the PepcDB database [14]. ParCrys predictions and data used for training and benchmarking are available at [77].

### 5.3 XANNpred

Structural proteomics consortia routinely apply sequence-based selection constraints on their targets, which influence the composition of the associated databases (e.g. PepcDB, TargetDB). With this in mind, a pair of algorithms named XANNpred-SG and XANNpred-PDB were respectively developed using data from PepcDB and the PDB [16]. The XANNpred algorithms utilise a large number of features for prediction, including dipeptide frequencies, predicted disorder [58], transmembrane regions [44] and secondary structure [61]. In contrast to ParCrys [14] and XtalPred [36], each of the XANNpred algorithms were robust to either predicting over data taken from the whole PDB or predicting over structural proteomics datasets (PepcDB) [16]. Additionally, XANNpred can generate windowed graphs of crystallisation propensity over a protein sequence in order to assist construct design. XANNpred predictions are available from [73]. Both XANNpred-SG and XANNpred-PDB were found to outperform other publicly available algorithms (PXS [28], XtalPred [36], OB-Score [15], ParCrys [14]) over several non-redundant blind test datasets [16]. Section 5.4, below extends this comparison to a recently published algorithm, PPCpred [72].

### 5.4 Evaluation of current methods to predict overall success of selected targets

**Fig. 2:**
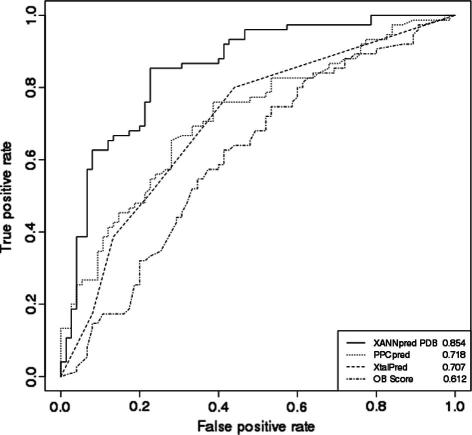
Comparison of methods for predicting overall success in the structure determination pipeline. This figure shows receiver operator characteristic (ROC) curves for the methods XANNpred-PDB, PPCpred, XtalPred and OB-Score on a non-redundant set of 150 proteins that were developed as an independent blind test for XANNpred-PDB [16]. Areas under the ROC curve are given in the bottom right-hand corner. XANNpred performs significantly better than the next best algorithm, PPCpred.

Fig. 2 gives comparison of the methods XANNpred-PDB [16], XtalPred [36], PPCpred [72] and OB-Score [15] on a nonredundant dataset of 150 proteins that were controlled to be an independent blind test of XANNpred-PDB performance [16]. Briefly, the blind test dataset includes 75 proteins from the PDB [69] and 75 proteins from PepcDB that had been cloned but where work was stopped before crystals were obtained [16]. Predictions for PPCpred were obtained from [74], data for the other algorithms were taken from [16]. XANNpred-PDB gave Matthew’s correlation coefficient (MCC) of 0.63 and area under the receiver operator characteristic curve (AROC) of 0.854, performing significantly better than the next best algorithm PPCpred (two-tailed *p* < 0.0091); PPCpred had AROC of 0.718 and best possible MCC of 0.37. XtalPred performs similarly to PPCpred (AROC 0.707, best possible MCC 0.37) followed by the OB-Score (AROC 0.612, best possible MCC 0.23). This test dataset was controlled by stringent approaches [16] to enable an independent blind test of XANNpred-PDB performance. However, estimates for the other algorithms (PPCpred XtalPred, OB-Score) are likely inflated due to some degree of overlap between their training data and the test data studied here. Even so, XANNpred performed best and appears to be the method of choice for estimating targets’ overall success in the structural biology pipeline.

## 6 Single point of reference resource for target selection and optimisation

As discussed above, numerous computational approaches are relevant to target selection and optimisation. The task of running these calculations, as well as integration, management and visualisation of the resultant information represents a significant challenge. Single point of reference resources have been developed in order to address these issues. The Oxford Protein Analysis Linker (OPAL) [4] was an early resource for this purpose, and collected information from several websites that performed individual analysis steps. However OPAL does not provide integration or storage of results. Other similar resources are also available but without a structural biology focus, such as ExPASy, Dasty3 and ANNIE [80–82]; analysis with these tools becomes very laborious over large numbers of alternative targets (e.g. orthologues, constructs). The XtalPred [36] website provides some level of results integration for relatively few algorithms, but does not include display of results on a multiple sequence alignment (MSA). Greater integration over a user-supplied MSA is offered by MACSIMS [83] which also propagates annotations by homology inference. However, MACSIMS is not focused on structural biology and no ranking of sequences is given. Also, MACSIMS returns a limited subset of annotation types and only annotation that is amenable to display on a MSA. The Target Optimisation Utility (TarO) [12], developed within the Scottish Structural Proteomics Facility (SSPF) has advantages over the above tools in that it provides for more sophisticated analysis, integration and visualisation of a large number of results.

### 6.1 The Target Optimisation Utility (TarO)

**Fig. 3:**
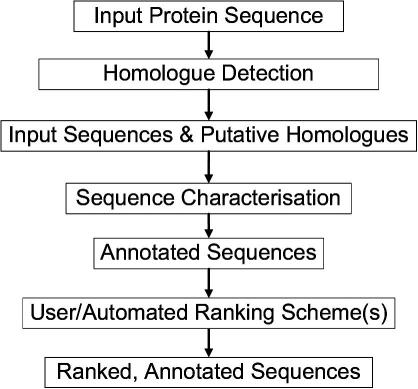
Outline of TarO workflow. This figure outlines the major steps involved in the TarO workflow. Protein input sequences provide the starting point for homologue searching. The input and all matched homologues are then annotated in the sequence characterisation step. An initial ranking is automatically provided within the user interface, but human analysis of the presented results is an important step.

**Fig. 4:**
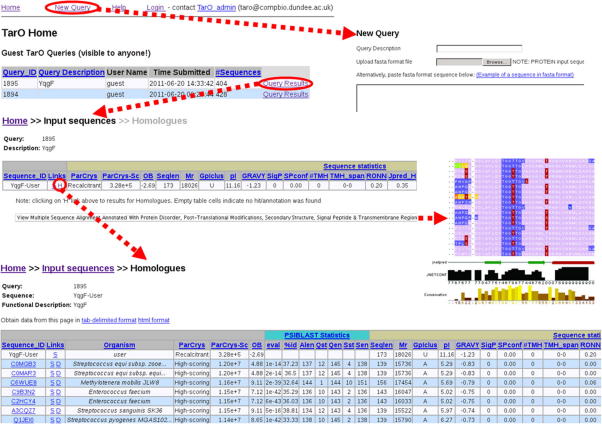
Key features of TarO user interface. This figure shows snapshots of several TarO user interface pages. Dashed arrows (red in online figure) indicate navigation by clicking on the relevant links. The TarO guest user ’Home’ page is shown at the top, clicking on the ’New Query’ link circled (red in online figure), navigates to the new query submission form; clicking on the ’Query Results’ link, circled (red in online figure), navigates to the ’Input Sequences’ page for the relevant query. Links on the ’Input Sequences’ page enable navigation to the homologues page (’H’), circled (red in online figure), and display of the multiple sequence alignment. Please note that the tables shown in this figure are truncated, and have many additional results columns.

TarO [12,84] takes a protein sequence(s) as input, and searches for homologues to generate a pool of potential alternative targets for structural work. The input and associated homologues are analysed in several annotation steps, and the results stored in a database. The TarO website provides an interface for access to results, integrating closely with the Jalview [85] program to visualise complex annotation over a multiple sequence alignment (MSA). The TarO workflow is outlined in Fig. 3 and key features of the user interface are summarised in Fig. 4. A guest account is available for unrestricted access to TarO and information about obtaining a private account for academic use is given at [86]. Guest queries are deleted from the server after a minimum of eight days. Login to a private account or navigating to the guest area displays a ‘Home’ page that summarises the submitted queries (Fig. 4). The ‘New Query’ link navigates to an easy to use web form for query submission, and a maximum of 20 sequences are accepted. The query submission form also gives the opportunity to specify the maximum number (default 100) of matched sequences included as input for MUSCLE [87] to generate the MSA. Ideally the MSA would include enough sequence diversity to enable identification of conserved residues, whilst excluding sequences unrelated in evolution and so generate a meaningful alignment. When multiple input sequences are submitted to TarO, we recommend that these are related (e.g. orthologues) in order to help produce a more useful MSA. From the ‘Home’ page, clicking on the ‘Query Results’ link navigates to the ‘Input sequences’ page, which includes a table to track progress of calculations in a ‘Query Status’ table according to a traffic lights system; orange shows the step has been initialised, green indicates completion and red means that the calculation failed. The ‘Input sequences’ page also summarises results for the input sequence(s) in a table (Fig. 4) and gives a link to display an annotated MSA in Jalview [85]. The Jalview full application enables DAS annotation lookup for the aligned sequences linking to significant additional information such as Gene Ontology terms [38]. Table column headings link to relevant parts of the help documentation, which provide more explanation of the information presented. This table gives various sequence statistics (e.g. molecular weight), including summaries of BLAST [88] searching TargetDB [68], PDB [69] and UniProt [78]. Links within this table enable display of further details including RPSBLAST search results and allow navigation to the Dasty and UniProt resources [81].

### 6.2 Exploring alternative homologues and constructs in TarO

In order to investigate alternative targets, clicking on the link labelled ‘H’ in the ‘Input Sequences’ table (Fig. 4) navigates to a page of annotated putative homologues tabulated and ranked by estimated crystallisation success (ParCrys [14]) and functional similarity (PSIBLAST expectation value [88]). Additional information relevant to estimating success in obtaining diffracting crystals is supplied, including sequence length, predicted transmembrane segments, secondary structure, and protein disorder. Results are presented from BLAST [88] searching the homologues against the PDB and TargetDB. In the results table, the “99%qcov” column shows a true/false value (i.e. 1/0) to indicate if the top BLAST hit covers 99% of the query (homologue) sequence; the “99%qcov + 99%id” column shows a true/false value (i.e. 1/0) to indicate if the query sequence has both 99% coverage and at least 99% sequence identity to the top hit. These indicators allow rapid evaluation of pre-existing work in structural genomics consortia on the target of interest, and reveal whether a high-resolution structural model has been deposited in the PDB. Annotated status of the matched target as recorded in TargetDB can be retrieved by clicking on the ‘T’ link in the results table, under the TargetDB section ‘More’ column; similar information for the PDB search is available by clicking on the ‘P’ link under the PDB Top Hit section ‘More’ column.

**Fig. 5:**
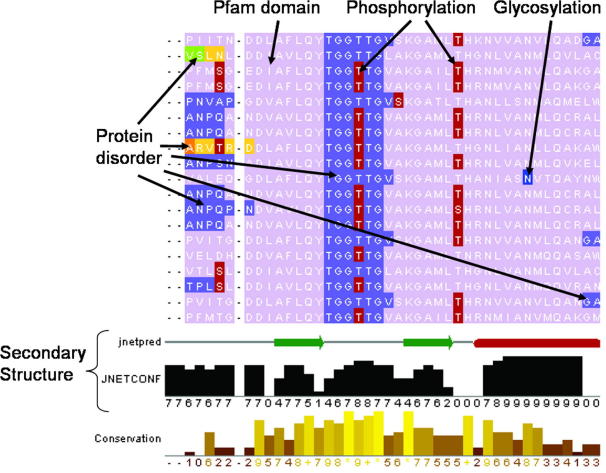
Annotated multiple sequence alignment. This figure shows a portion of an annotated multiple sequence alignment, visualised with Jalview [85]. The different shades (colours in online figure) on the aligned sequences represent different annotation types. The lightest grey (lilac) corresponds to a Pfam domain. Predicted GlobPlot [60] and Disembl [59] disorder are show in medium greys (slate blue, light/dark orange, green). Predicted post-translational modifications (PTMs), phosphorylation [67] and N-linked glycosylation [90] are respectively shown in dark grey (red) and medium grey (blue). Jpred [61] predicted secondary structure for the input sequence is shown on the line entitled ‘jnetpred’ that runs towards the bottom of the figure. Related annotations are grouped and may be selectively displayed in order to enable visualisation and interpretation of the information. The TarO annotation groupings are viewed inside the Jalview ‘Features Settings’ box. For example, Disembl and GlobPlot disorder are grouped together, whilst Pfam domains and RONN disorder are in a separate group. There is also a group for protein disorder predicted by Disembl and RONN. From the ‘Feature Settings’ box, the user can change the display of the various groups in order to customise the presence or absence of annotations on the MSA. The order of annotations displayed is also specified within the ‘Feature Settings’ box. For example the annotation layer for PTMs is best displayed on top of the other annotations in this figure. Therefore the medium grey (slate blue) GlobPlot disorder annotation on the sequence region ‘TGGTTG’ is displayed underneath the dark grey (red) predicted phosphorylation site annotation on the second threonine residue of the ‘TGGTTG’ sequence. The row at the bottom of the figure shows the alignment conservation and is automatically calculated by Jalview.

As part of assessing targets’ functional similarity we recommend manually inspecting the patterns of sequence annotation and conserved residues across a multiple sequence alignment (MSA). TarO generates an annotated MSA from the input sequence and top-scoring putative homologues, aligning with the MUSCLE algorithm [87]. Fig. 5 shows part of a MSA produced by TarO, and viewed in Jalview [85]. Please note that in order to access the Jalview applet from TarO, a correctly installed Java Runtime Environment is required. As well as providing for assessment of functional similarity, this MSA is useful in construct design for target optimisation. Annotated features include predicted protein domains [62,89], secondary structure [61], post-translational modifications (PTMs) [65,67,90], signal peptide [91], transmembrane regions [44], and disorder [58–60] (Fig. 5). Of course construct optimisation generally seeks to minimise unfavourable features in the target protein (e.g. disorder, signal peptide, transmembrane regions). Design of truncated constructs, for example to remove disordered N- or C-termini or to isolate a domain(s) requires careful inspection of predicted protein disorder and domain boundaries and should avoid disruption of any secondary structure element. Inspection of the MSA annotated with results from several disorder prediction algorithms enables a consensus view over the aligned sequences, which is helpful for determining construct boundaries. Where no domains are found by database searching, one practical strategy for N- or C-terminal truncation to improve crystallisation success is to conservatively remove any continuous region of predicted disordered sequence from the terminus up to the start of the first predicted secondary structure element, possibly testing several constructs with alternate boundaries. The annotated MSA is also useful as a sequence analysis tool, assisting identification of conserved functional residues.

Different visualisations of sequence features can be selected using the Jalview [85] ‘Feature Settings’ window that appears when the MSA is displayed. It is important to note that the MSA display requires some initial adjustment to ensure visibility of all sequence features. For example, some fraction of annotated post-translational modifications (and signal peptide) is frequently hidden underneath other annotation (e.g. domains); therefore we strongly recommend ensuring all annotations are visible, which is done by unchecking and rechecking the ‘PTMs_ + _SignalP’ tick-box at the top of the Feature Settings window. The same is true for transmembrane regions (‘TM_regions’ tick-box). By default the MSA display initialises to show domains (Pfam [62]), disorder (RONN [58]), post-translational modifications (various), signal peptide (SignalP [91]), and transmembrane regions (TMHMM2 [44]); however, additional disorder annotations can be shown by checking the appropriate boxes in the Feature Settings window. A key practical consideration is that annotations for the most recently checked tick-box will be always be displayed on top of all other annotations. Further discussion and recommendations for the annotated MSA are given at [92]. TarO also supplies information relevant to particular pipeline stages. For example frequencies of key amino acids are given, including Cys and Met which are relevant to solubility as well as phasing by anomalous dispersion. A tutorial is also available from the TarO website, please contact us (taro@compbio.dundee.ac.uk) for login details.

## 7 Concluding remarks

Some of the current approaches in target selection and optimisation were discussed, as well as how these approaches can mitigate the non-trivial task of successfully navigating the various stages in the structural proteomics pipeline. The tools TarO [12] and crystallisation propensity predictors (OB-Score, ParCrys, XANNpred) [14–16] were employed within the Scottish Structural Proteomics Facility (SSPF) and partly contributed to a good rate of success where 61 (21%) of the 295 targets taken into expression trials lead to diffraction-quality crystals [3]. Indeed, the XANNpred predictor was found to outperform other available methods on independent blind test data [16] including PPCpred [72], PXS [28] and XtalPred [36,76]. Combining experimental measurements and protein sequence information (e.g. [93]) is an interesting approach. However experimental characterisation requires purified protein, therefore predictions would be focused on crystal growth and not available for decision-making during initial target selection. Also, we have highlighted benefits in selecting targets with a single algorithm to predict successful progression through all stages leading to a high-resolution structural model, rather than combining multiple results arising from different predictors for each key stage of the structural proteomics pipeline.

## Figures and Tables

**Table 1:** Estimation of protein characteristics useful for target selection and optimisation.

Protein characteristics	Exemplar algorithms and/or databases
Homology relationships	Algorithms: BLAST[88], SCANPS [95], MUSCLE [87], Magicmatch [96]
	Databases: eggNOG [55], InParanoid [56], UniProt [78]
Matches to known structures/declared targets	PDB [69], TargetDB/PepcDB [68]
Domains	Algorithms: HMMER [97], RPSBLAST Databases: Pfam [62], CDD [63], SMART [98], Superfamily [99], Biozon [100]
Protein interactions	PIPS [51], STRING [101]
Disorder/low-complexity sequence	Disembl [59], RONN [58], GlobPlot [60], SEG [102]
Signal peptide and transmembrane regions	SignalP [91], Phobius [45], TMHMM2 [44]
Glycosylation sites	NetOGlyc [65], NetNGlyc [90]
Phosphorylation sites	NetPhos [67], Musite [103]
Secondary structure	JPred [61], PSIPRED [104]
Surface entropy	SERp [9]
Chemical properties: isoelectric point (pI), molecular weight, charge, sequence length, extinction coefficient, #Methionines, #Cysteines, #Histidines, hydrophobicity, protease sites	Bioperl [105], PEPSTATS (EMBOSS) [106]
Annotated function	Gene Ontology [38]
Overall tractability (selected to diffraction-quality crystals)	XANNPred [16], XtalPred [36], OB-Score [15], ParCrys [14]

## References

[1] Chruszcz M., Domagalski M., Osinski T., Wlodawer A., Minor W. (2010). Curr. Opin. Struct. Biol..

[2] Xiao R., Anderson S., Aramini J., Belote R., Buchwald W.A., Ciccosanti C. (2010). J. Struct. Biol..

[3] Oke M., Carter L.G., Johnson K.A., Liu H., McMahon S.A., Yan X. (2010). J. Struct. Funct. Genomics.

[4] Albeck S., Alzari P., Andreini C., Banci L., Berry I.M., Bertini I. (2006). Acta Crystallogr. D.

[5] Bravo J., Aloy P. (2006). Curr. Opin. Struct. Biol..

[6] Bray J.E., Marsden R.L., Rison S.C.G., Savchenko A., Edwards A.M., Thornton J.M. (2004). Bioinformatics.

[7] Chandonia J.-M., Brenner S.E. (2005). Proteins.

[8] Chandonia J.-M., Kim S.-H., Brenner S.E. (2006). Proteins.

[9] Goldschmidt L., Cooper D.R., Derewenda Z.S., Eisenberg D. (2007). Protein Sci..

[10] Leulliot N., Trésaugues L., Bremang M., Sorel I., Ulryck N., Graille M. (2005). Acta Crystallogr. D Biol. Crystallogr..

[11] Manjasetty B.A., Turnbull A.P., Panjikar S., Büssow K., Chance M.R. (2008). Proteomics.

[12] Overton I.M., van Niekerk C.A.J., Carter L.G., Dawson A., Martin D.M.A., Cameron S. (2008). Nucleic Acids Res..

[13] Pache R.A., Aloy P. (2008). Proteomics.

[14] Overton I.M., Padovani G., Girolami M.A., Barton G.J. (2008). Bioinformatics.

[15] Overton I.M., Barton G.J. (2006). FEBS Lett..

[16] Overton I.M., van Niekerk C.A.J., Barton G.J. (2011). Proteins.

[17] International Structural Genomics Organisation (ISGO) List of Structural Genomics and Proteomics Projects, <http://www.isgo.org/list/index.php>.

[18] Lesley S.A., Kuhn P., Godzik A., Deacon A.M., Mathews I., Kreusch A. (2002). Proc. Natl. Acad. Sci. USA.

[19] Quevillon-Cheruel S., Collinet B., Zhou C.-Z., Minard P., Blondeau K., Henkes G. (2002). J. Synchrotron Rad..

[20] Angov E., Hillier C.J., Kincaid R.L., Lyon J.A. (2008). PLoS One.

[21] Ioerger T.R., Sacchettini J.C. (2009). Curr. Opin. Microbiol..

[22] Cascante M., Boros L.G., Comin-Anduix B., de Atauri P., Centelles J.J., Lee P.W.-N. (2002). Nat. Biotechnol..

[23] Dessailly B.H., Nair R., Jaroszewski L., Fajardo J.E., Kouranov A., Lee D. (2009). Structure.

[24] Membrane Protein Structure Initiative (MPSI) Homepage, <http://www.mpsi.ac.uk/>.

[25] Punta M., Love J., Handelman S., Hunt J.F., Shapiro L., Hendrickson W.A. (2009). J. Struct. Funct. Genomics.

[26] Canaves J.M., Page R., Wilson I.A., Stevens R.C. (2004). J. Mol. Biol..

[27] Goh C.-S., Lan N., Douglas S.M., Wu B., Echols N., Smith A. (2004). J. Mol. Biol..

[28] Price W.N., Chen Y., Handelman S.K., Neely H., Manor P., Karlin R. (2009). Nat. Biotechnol..

[29] Smialowski P., Martin-Galiano A.J., Mikolajka A., Girschick T., Holak T.A., Frishman D. (2007). Bioinformatics.

[30] Smialowski P., Schmidt T., Cox J., Kirschner A., Frishman D. (2006). Proteins: Struct. Funct. Bioinf..

[31] Eisenhaber B., Eisenhaber F. (2007). Curr. Protein Pept. Sci..

[32] Idicula-Thomas S., Kulkarni A.J., Kulkarni B.D., Jayaraman V.K., Balaji P.V. (2006). Bioinformatics.

[33] Jones S., Thornton J.M. (1996). Proc. Natl. Acad. Sci..

[34] Tsai C., Lin S.L., Wolfson H.J., Nussinov R. (1997). Protein Sci..

[35] Derewenda Z.S., Vekilov P.G. (2006). Acta Crystallogr. D Biol. Crystallogr..

[36] Slabinski L., Jaroszewski L., Rychlewski L., Wilson I.A., Lesley S.A., Godzik A. (2007). Bioinformatics.

[37] Magnan C.N., Randall A., Baldi P. (2009). Bioinformatics.

[38] Ashburner M., Ball C.A., Blake J.A., Botstein D., Butler H., Cherry J.M. (2000). Nat. Genet..

[39] Wallin E., von Heijne G. (1998). Protein Sci..

[40] Raman P., Cherezov V., Caffrey M. (2006). Cell. Mol. Life Sci..

[41] Membrane Protein Data Bank (MPDB), <http://www.mpdb.tcd.ie/>.

[42] Lee J.K., Stroud R.M. (2010). Curr. Opin. Struct. Biol..

[43] Wagner S., Baars L., Ytterberg A.J., Klussmeier A., Wagner C.S., Nord O. (2007). Mol. Cell. Proteomics.

[44] Krogh A., Larsson B., von Heijne G., Sonnhammer E.L. (2001). J. Mol. Biol..

[45] Kall L., Krogh A., Sonnhammer E.L.L. (2007). Nucleic Acids Res..

[46] Mohan A., Uversky V.N., Radivojac P. (2009). PLoS Comput. Biol..

[47] He B., Wang K., Liu Y., Xue B., Uversky V.N., Dunker A.K. (2009). Cell Res..

[48] Dunker A.K., Gough J. (2011). Curr. Opin. Struct. Biol..

[49] SPINE 2 – COMPLEXES from Receptor to Gene: Structures of Complexes, <http://www.spine2.eu/SPINE2/>.

[50] Chatr-aryamontri A., Ceol A., Palazzi L.M., Nardelli G., Schneider M.V., Castagnoli L. (2007). Nucleic Acids Res..

[51] McDowall M.D., Scott M.S., Barton G.J. (2009). Nucleic Acids Res..

[52] Yamasaki C., Takeda Jun-ichi, Habara T., Ogawa M., Noda A., Sakate T. (2008). Nucleic Acids Res..

[53] Hunter S., Apweiler R., Attwood T.K., Bairoch A., Bateman A., Binns D. (2009). Nucleic Acids Res..

[54] Rost B. (1999). Protein Eng..

[55] Muller J., Szklarczyk D., Julien P., Letunic I., Roth A., Kuhn M. (2010). Nucleic Acids Res..

[56] Östlund G., Schmitt T., Forslund K., Köstler T., Messina D.N., Roopra S. (2010). Nucleic Acids Res..

[57] Wong W.-C., Maurer-Stroh S., Eisenhaber F. (2010). PLoS Comput. Biol..

[58] Yang Z.R., Thomson R., McNeil P., Esnouf R.M. (2005). Bioinformatics.

[59] Linding R., Jensen L.J., Diella F., Bork P., Gibson T.J., Russell R.B. (2003). Structure.

[60] Linding R., Russell R.B., Neduva V., Gibson T.J. (2003). Nucleic Acids Res..

[61] Cole C., Barber J.D., Barton G.J. (2008). Nucleic Acids Res..

[62] Finn R.D., Mistry J., Tate J., Coggill P., Heger A., Pollington J.E. (2010). Nucleic Acids Res..

[63] Marchler-Bauer A., Anderson J.B., Chitsaz F., Derbyshire M.K., DeWeese-Scott C., Fong J.H. (2009). Nucleic Acids Res..

[64] Attwood T.K., Bradley P., Flower D.R., Gaulton A., Maudling N., Mitchell A.L. (2003). Nucleic Acids Res..

[65] Julenius K., Mølgaard A., Gupta R., Brunak S. (2005). Glycobiology.

[66] Maurer-Stroh S., Koranda M., Benetka W., Schneider G., Sirota F.L., Eisenhaber F. (2007). PLoS Comput. Biol..

[67] Blom N., Gammeltoft S., Brunak S. (1999). J. Mol. Biol..

[68] Chen L., Oughtred R., Berman H.M., Westbrook J. (2004). Bioinformatics.

[69] Rose P.W., Beran B., Bi C., Bluhm W.F., Dimitropoulos D., Goodsell D.S. (2010). Nucleic Acids Res..

[70] Morris C., Pajon A., Griffiths S.L., Daniel E., Savitsky M., Lin B. (2011). Acta Crystallogr. D Biol. Crystallogr..

[71] Babnigg G., Joachimiak A. (2010). J. Struct. Funct. Genomics.

[72] Mizianty M.J., Kurgan L. (2011). Bioinformatics.

[73] XANNpred Home Page, <http://www.compbio.dundee.ac.uk/xannpred>.

[74] PPCpred Server Home, <http://biomine-ws.ece.ualberta.ca/PPCpred.html>.

[75] XtalPred Server: Home, <http://ffas.burnham.org/XtalPred-cgi/xtal.pl>.

[76] Slabinski L., Jaroszewski L., Rodrigues A.P.C., Rychlewski L., Wilson I.A., Lesley S.A. (2007). Protein Sci..

[77] ParCrys and OB-Score Home Page, <http://www.compbio.dundee.ac.uk/xtal>.

[78] The UniProt Consortium (2010). Nucleic Acids Res..

[79] OB-Score Software Download, <http://www.compbio.dundee.ac.uk/obscore/>.

[80] ExPASy Proteomics Server, <http://expasy.org/>.

[81] Jones P., Vinod N., Down T., Hackmann A., Kahari A., Kretschmann E. (2005). Bioinformatics.

[82] Ooi H.S., Kwo C.Y., Wildpaner M., Sirota F.L., Eisenhaber B., Maurer-Stroh S. (2009). Nucleic Acids Res..

[83] Thompson J.D., Muller A., Waterhouse A., Procter J., Barton G.J., Plewniak F. (2006). BMC Bioinf..

[84] TarO_v2.5, <http://www.compbio.dundee.ac.uk/taro/cgi-taro/v3_targpipe_input.pl>.

[85] Waterhouse A.M., Procter J.B., Martin D.M.A., Clamp M., Barton G.J. (2009). Bioinformatics.

[86] TarO Registration Details, <http://www.compbio.dundee.ac.uk/taro/TarORegistration.htm>.

[87] Edgar R.C. (2004). Nucleic Acids Res..

[88] Altschul S.F., Madden T.L., Schäffer A.A., Zhang J., Zhang Z., Miller W. (1997). Nucleic Acids Res..

[89] Letunic I., Doerks T., Bork P. (2009). Nucleic Acids Res..

[90] NetNGlyc 1.0 Server, <http://www.cbs.dtu.dk/services/NetNGlyc/>.

[91] Bendtsen J.D., Nielsen H., von Heijne G., Brunak S. (2004). J. Mol. Biol..

[92] TarO Help: Annotated Multiple Sequence Alignment Section, <http://www.compbio.dundee.ac.uk/taro/TarO_help.html#MSA>.

[93] Zucker F.H., Stewart C., dela Rosa J., Kim J., Zhang L., Xiao L. (2010). J. Struct. Biol..

[94] Davidsen T., Beck E., Ganapathy A., Montgomery R., Zafar N., Yang Q. (2009). Nucleic Acids Res..

[95] Walsh T.P., Webber C., Searle S., Sturrock S.S., Barton G.J. (2008). Nucleic Acids Res..

[96] Smith M., Kunin V., Goldovsky L., Enright A.J., Ouzounis C.A. (2005). Bioinformatics.

[97] Finn R.D., Clements J., Eddy S.R. (2011). Nucleic Acids Res..

[98] Ponting C.P., Schultz J., Milpetz F., Bork P. (1999). Nucleic Acids Res..

[99] Wilson D., Madera M., Vogel C., Chothia C., Gough J. (2007). Nucleic Acids Res..

[100] Birkland A. (2006). Nucleic Acids Res..

[101] Jensen L.J., Kuhn M., Stark M., Chaffron S., Creevey C., Muller J. (2009). Nucleic Acids Res..

[102] Wootton J.C., Federhen S. (1993). Comput. Chem..

[103] Gao J., Thelen J.J., Dunker A.K., Xu D. (2010). Mol. Cell. Proteomics.

[104] McGuffin L.J., Bryson K., Jones D.T. (2000). Bioinformatics.

[105] Stajich J.E., Block D., Boulez K., Brenner S.E., Chervitz S.A., Dagdigian C. (2002). Genome Res..

[106] Olson S.A. (2002). Brief. Bioinf..

